# Supplementary material for: Optogenetic control of receptors reveals distinct roles for actin- and Cdc42-dependent negative signals in chemotactic signal processing
Source: Nat Commun. 2021 Nov 16;12:6148. doi: 10.1038/s41467-021-26371-z (PMC8595684; doi:10.1038/s41467-021-26371-z)
Supplement: Supplementary file 1 — Supplementary Information [file 41467_2021_26371_MOESM1_ESM.pdf]

Supplementary Information:

Supplementary Figure 1

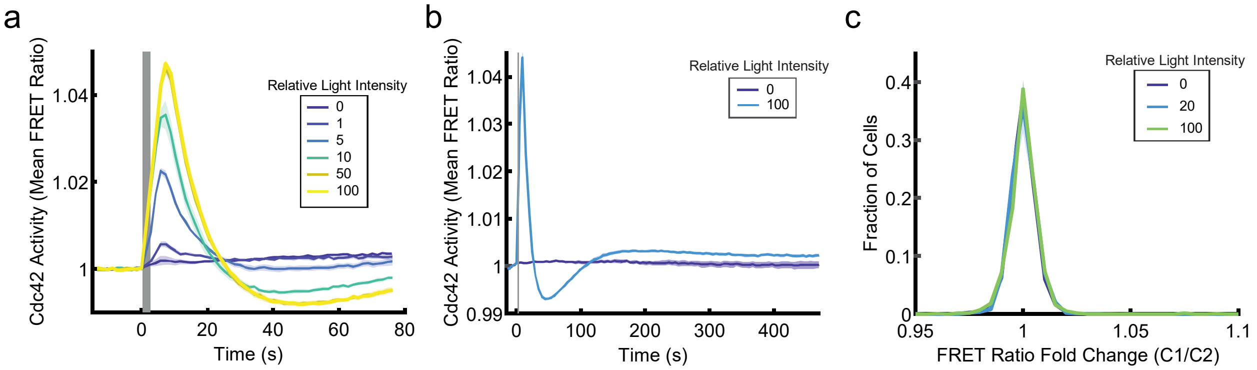

Supplementary Figure 1: Dose-dependent positive and negative signals downstream of receptors shape a graded Cdc42 response. (**a**) Subset of Cdc42-dose response curves, highlighting the graded nature of the Cdc42 response. Data are presented as mean ± s.e.m. ($n_{well\ replicates}$ = 23 for Relative Light Intensity=0, $n_{well\ replicates}$ = 7 for Relative Light Intensity=1, $n_{well\ replicates}$ = 7 for Relative Light Intensity=5, $n_{well\ replicates}$ = 12 for Relative Light Intensity=10, $n_{well\ replicates}$ = 7 for Relative Light Intensity=50, $n_{well\ replicates}$ = 7 for Relative Light Intensity=100). (**b**) Single stimulation experiment with extended duration captures late phase oscillatory behavior as the Cdc42 response adapts to a baseline level. Data are presented as mean ± s.e.m. ($n_{well\ replicates}$ = 11 for each stimulation condition). (**c**) Control for fraction of cells responding to light stimulus strength. Control for FRET ratio fold change was calculated by taking the ratio of the control window (C1) to the control window (C2). Data are presented as mean ± s.e.m. ($n_{experiment}$ = 4). Across the four experiments, n = 4127 cells (relative light intensity = 0), n = 2317 cells (relative light intensity = 20), n = 2261 cells (relative light intensity =100). Source data are provided as a Source Data file.

Supplementary Figure 2

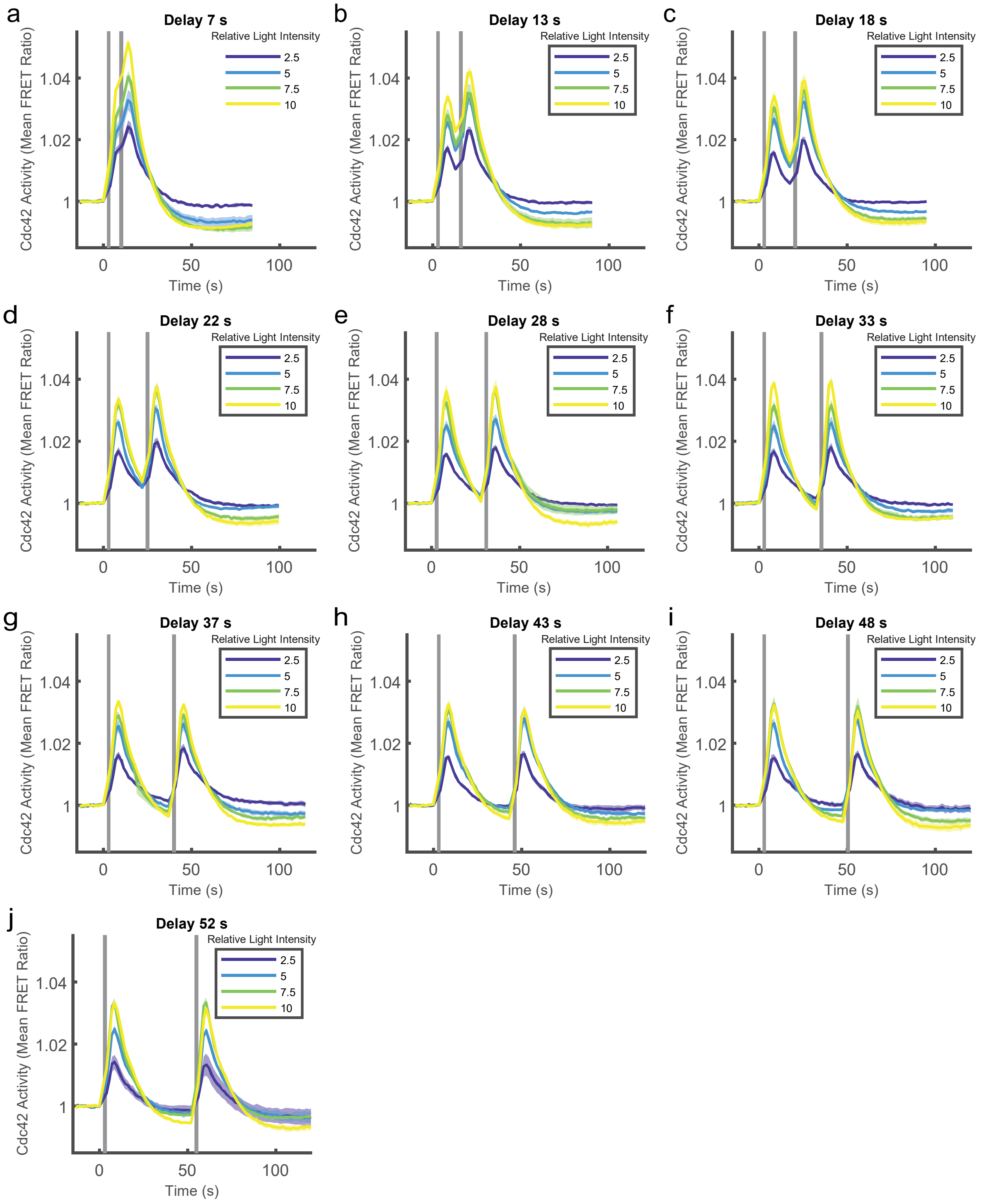

Supplementary Figure 2: Responses to sequential stimuli are independent. (**a-j**) Cdc42 response to two equal duration and light intensity stimulations with varying time delays. (**a**) Data are presented as mean ± s.e.m. ($n_{well\ replicates}$ = 6 (Relative light intensity = 2.5), $n_{well\ replicates}$ = 6 (Relative light intensity = 5), $n_{well\ replicates}$ = 6 (Relative light intensity = 7.5), $n_{well\ replicates}$ = 30 (Relative light intensity = 10)). (**b**) Data are presented as mean ± s.e.m. ($n_{well\ replicates}$ = 7 (Relative light intensity = 2.5), $n_{well\ replicates}$ = 6 (Relative light intensity = 5), $n_{well\ replicates}$ = 6 (Relative light intensity = 7.5), $n_{well\ replicates}$ = 6 (Relative light intensity = 10)). (**c**) Data are presented as mean ± s.e.m. ($n_{well\ replicates}$ = 30 (Relative light intensity = 2.5), $n_{well\ replicates}$ = 7 (Relative light intensity = 5), $n_{well\ replicates}$ = 6 (Relative light intensity = 7.5), $n_{well\ replicates}$ = 6 (Relative light intensity = 10)). (**d**) Data are presented as mean ± s.e.m. ($n_{well\ replicates}$ = 6 (Relative light intensity = 2.5), $n_{well\ replicates}$ = 30 (Relative light intensity = 5), $n_{well\ replicates}$ = 7 (Relative light intensity =7.5), $n_{well\ replicates}$ = 6 (Relative light intensity = 10)). (**e**) Data are presented as mean ± s.e.m. ($n_{well\ replicates}$ = 6 (Relative light intensity = 2.5), $n_{well\ replicates}$ = 6 (Relative light intensity = 5), $n_{well\ replicates}$ = 30 (Relative light intensity = 7.5), $n_{well\ replicates}$ = 6 (Relative light intensity = 10)). (**f**) Data are presented as mean ± s.e.m. ($n_{well\ replicates}$ = 5 (Relative light intensity = 2.5), $n_{well\ replicates}$ = 5 (Relative light intensity = 5), $n_{well\ replicates}$ = 5 (Relative light intensity =7.5), $n_{well\ replicates}$ = 26 (Relative light intensity = 10)). (**g**) Data are presented as mean ± s.e.m. ($n_{well\ replicates}$ = 5 (Relative light intensity = 2.5), $n_{well\ replicates}$ = 4 (Relative light intensity = 5), $n_{well\ replicates}$ = 4 (Relative light intensity = 7.5), $n_{well\ replicates}$ = 4 (Relative light intensity = 10)). (**h**) Data are presented as mean ± s.e.m. ($n_{well\ replicates}$ = 26 (Relative light intensity = 2.5), $n_{well\ replicates}$ = 5 (Relative light intensity =5), $n_{well\ replicates}$ = 4 (Relative light intensity = 7.5), $n_{well\ replicates}$ = 4 (Relative light intensity = 10)). (**i**) Data are presented as mean ± s.e.m. ($n_{well\ replicates}$ = 4 (Relative light intensity = 2.5), $n_{well\ replicates}$ = 26 (Relative light intensity = 5), $n_{well\ replicates}$ = 5 (Relative light intensity = 7.5), n_{well}

$_{replicates}$ = 4 (Relative light intensity = 10)). (**j**) Data are presented as mean ± s.e.m. ($n_{well\ replicates}$ = 4 (Relative light intensity = 2.5), $n_{well\ replicates}$ = 4 (Relative light intensity = 5), $n_{well\ replicates}$ = 26 (Relative light intensity = 7.5), $n_{well\ replicates}$ = 4 (Relative light intensity = 10)). Source data are provided as a Source Data file.

Supplementary Figure 3

a

Forward sequencing results

	Synthetic sgRNA #1		Synthetic sgRNA #2	# of additional bp not matching ref sequence
Reference Sequence	ATTTTTCTTTTTTCTAGGGCAAGAGG	. . .	ATTTGAAAACGTGAAAGAAAAGGTAAGCTGATCAGA	
19026 reads	ATTTTTCTTTTTTCTAGGGC------	. . .	----------------GAAAAGGTAAGCTGATCAGA	0
208 reads	ATTTTTCTTTTTTCTAGGGC------	. . .	----------------GAAAAGGTAAGCTGATCAGA	1
112 reads	ATTTTTCTTTTTTCTAGGGC------	. . .	----------------GAAAAGGTAAGCTGATCAGA	1

(79 bases omitted)

Reverse sequencing results

Reference Sequence	ATTTTTCTTTTTTCTAGGGCAAGAGG	. . .	ATTTGAAAACGTGAAAGAAAAGGTAAGCTGATCAGA	
17915 reads	ATTTTTCTTTTTTCTAGGGC------	. . .	----------------GAAAAGGTAAGCTGATCAGA	0
88 reads	ATTTTTCTTTTTTCTAGGGC------	. . .	----------------GAAAAGGTAAGCTGATCAGA	1
87 reads	ATTTTTCTTTTTTCTAGGGC------	. . .	----------------GAAAAGGTAAGCTGATCAGA	2

(79 bases omitted)

b

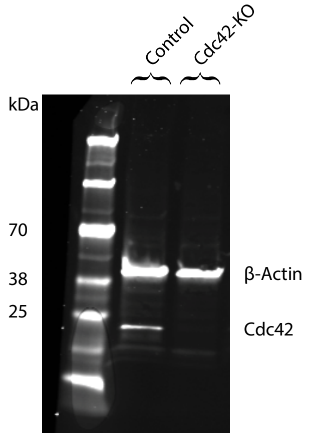

Supplementary Figure 3: The *CDC42* knockout is homozygous. (**a**) Amplicon sequencing results. The dominant sequence was identical for both forward and reverse primers. The single dominant read indicates that the same deletion is present for both alleles. (**b**) Full-lane western blot. Top bands correspond to an anti-β-Actin antibody while bottom band is reactive to an anti-Cdc42 antibody. Blot is representative of n = 3 experiments. Source data are provided as a Source Data file.

Supplementary Figure 4

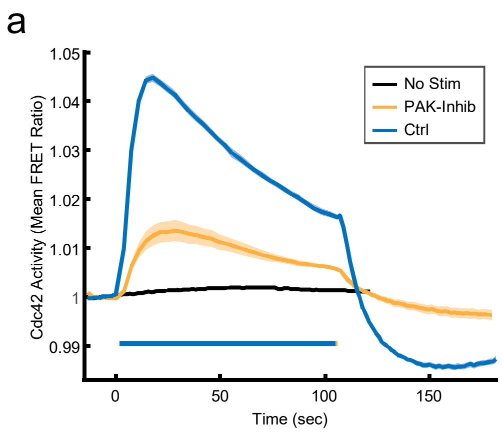

Supplementary Figure 4: Inhibition of PAK1 reduces Cdc42 response magnitude regardless of stimulation light power. (**a**) Control and PAK1-Inhibited cells responding to medium power light stimulation (Relative light intensity = 10). Data are presented as mean ± s.e.m. ($n_{well\ replicates}$ = 65 for non-stimulated, $n_{well\ replicates}$ = 59 for control and $n_{well\ replicates}$ = 16 for PAK1 inhibited cells). Source data are provided as a Source Data file.

Supplementary Figure 5

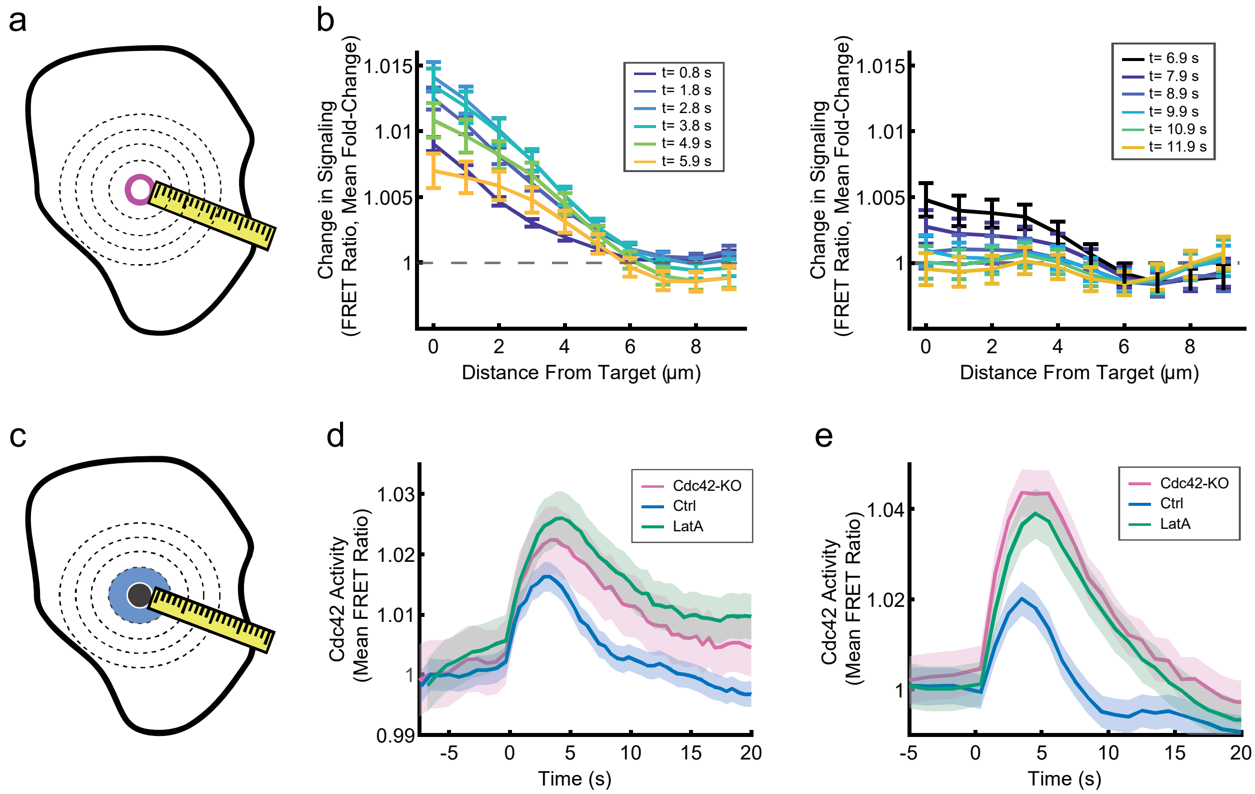

Supplementary Figure 5: The Cdc42-KO and Latrunculin-A perturbations prolong the duration of the Cdc42 response. (**a**) Schematic for center stimulation experiment analysis strategy. Cell pixels were aggregated based on their distance from the stimulus target site (magenta circle) for each frame in the experiment. (**b**) Time course encompassing activation and attenuation of Cdc42 response to single pulse center stimulation experiment. Relative Cdc42 response as a function of distance from the stimulation target site for control cells. The response attenuates by ~10 seconds post stimulation (Right panel). Experimental stimulation condition: one, 0.8 µW light-pulse with 10ms duration. Data are presented as mean ± s.e.m. (n = 181 cells). (**c**) Center stimulation experiment schematic. Pixels < 1µm from the stimulation site (blue shaded region) were used to generate plots in (**d-e**). (**d**) Cdc42 activity as a function of time for control, Cdc42-KO, and Latrunculin-A treated cells responding to one, 0.8 µW light-pulse with 10 ms duration.

Data are presented as mean ± s.e.m. (n = 181 cells for control, n = 67 for Cdc42-KO, and n = 175 for Latrunculin-A). (**e**) Cdc42 activity as a function of time for control, Cdc42-KO, and Latrunculin-A treated cells responding to one, 4.3 μW light-pulse with 10 ms duration. Data are presented as mean ± s.e.m. (n = 131 cells for control, n = 43 for Cdc42-KO, and n = 105 for Latrunculin-A-treated cells). Source data are provided as a Source Data file.

Supplementary Figure 6

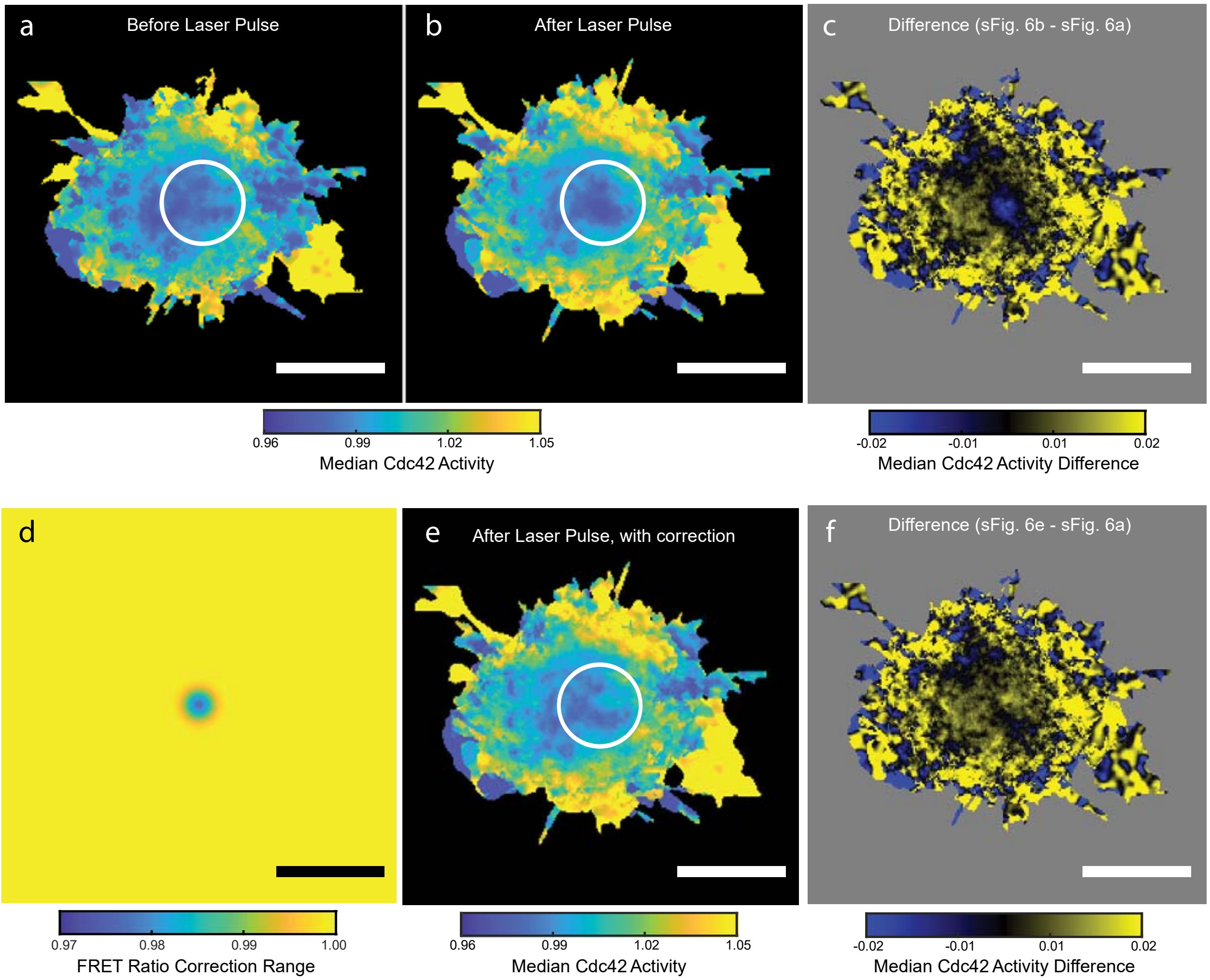

Supplementary Figure 6: Photobleaching correction for bleaching due to FRAP laser stimulation. (**a-b**) Median Cdc42 FRET ratio of n = 79 cells for the image immediately preceding (**a**) and succeeding (**b**) stimulation. Cells were stimulated with a strong, 37 μW, 10 ms laser pulse to maximize bleaching for computing the correction. White circles indicate the FRAP target region. Photobleaching causes a reduction in FRET signal (**b**). (**c**) Difference between post-stimulus and pre-stimulus image. (**d**) Photobleaching correction image for the frame immediately succeeding FRAP stimulation. (**e**) Median Cdc42 FRET ratio of n = 79 cells where

the photobleaching correction was applied to the post-stimulation frame. (**f**) Difference between photobleaching corrected post-stimulus and pre-stimulus images. Scale bar for all panels, 15 μm.

Supplementary Table 1

	Primer #	Primer Name	Sequence 5' to 3'
Cdc42 TomKat FRET Sensor Plasmid	p-01	tdTom_4tdKatgib.FOR	AGCAAACTGGGGCACAGCCTCGAGTCCGGACGCGGCCGCTCAATGGTGAGCAAGGGCGAG
	p-02	tdTom_4tdKatgib.REV	TATGGCTGATTATGATCTGGATCCAGAGTCGCTCCCTTGTACAGCTCGTCCATGCCGTAC
	p-03	PBSR_TomKatPrimed.FOR	tggcaaagaattGGGATCCGTCCGCTAGCGCTACCGGT
	p-04	PBSR_TKPrimed_ECORI.REV	taccatctttgctcatcttGCaGAATTCATGATCTGGATCCAGAGTCGCt
	p-05	PBSR_4TomKatTry2_For	TGTACAAGggaGCGACTCTGaagatgagcaaagatggtaaaaagaagaaaaagaagtcaa
	p-06	PBSR_4TomKatTry2_Rev	CATGGTGGCGACCGGTAGCGCGGATCCCaattctttgccaaaa
Parapinopsina plasmid	p-07	ProlacPP1_4SC160_F	tccatttcaggtgtcgtgaggatccACGAGAATGGACTCTAAGGGCAGCAGCCAG
	p-08	ProPP1_4mCit-SC321_R	ccttgctcacATGCATTTCAGCAGGGGCCACCTGG
	p-09	mCit_4ProPP1-SC321_F	CCCTGCTGAAATGCATgtgagcaagggcgaggagctg
	p-10	mCit_4PP1-Sc160_R	ctctagagcggccgcTCActtgtacagctcgtccatgccgag
	p-11	SC160_vect_F	gcggccgctctagagtcgac
	p-12	SC160_vectV2_R	TCTCGTggatcctcacgacacctgaaatg
	p-13	mCit_seq_F	gcaccatcttcttcaaggac
	p-14	mCit_Seq_R	gtccttgaagaagatggtgc
	p-15	PP1hs_Col_F	CTGTGGCCGTGATTGCCCTC
	p-16	PP1hs_Col_R	AGCCCTAGCAGCGGACCC
CRISPR Guides	p-17	Synthego Cdc42 Guide #1	tttcttttttctagGGCAAG
	p-18	Synthego Cdc42 Guide #2	ATTTGAAAACGTGAAAGAAA
Cdc42 KO amplicon sequencing	p-19	Cdc42 Amplicon Seq F	ACACTCTTTCCCTACACGACGCTCTTCCGATCTccagcatgcttttaacactttgagg
	p-20	Cdc42 Amplicon Seq R	GACTGGAGTTCAGACGTGTGCTCTTCCGATCTgaaaggagtctttggacagtggtg
Cdc42 KO Sanger sequencing	p-21	Cdc42Genomic-Seq_F (sanger seq)	tgttctgagtgcttgaag
	p-22	Cdc42Genomic-Seq_R (sanger seq)	AAAGGAGTCTTTGGACAG

Supplementary Table 1. List of primers used in this study.
