# Supplementary material for: Optogenetic control of receptors reveals distinct roles for actin- and Cdc42-dependent negative signals in chemotactic signal processing
Source: Nat Commun. 2021 Nov 16;12:6148. doi: 10.1038/s41467-021-26371-z (PMC8595684; doi:10.1038/s41467-021-26371-z)
Supplement: Supplementary file 2 — Description of Additional Supplementary Files [file 41467_2021_26371_MOESM2_ESM.pdf]

Description of Additional Supplementary Files

File name: Supplementary Movie 1

Description: Global stimulation of parapinopsinainduced Cdc42 activity is dependent on UV light stimulation and the presence of the 9-cis-retinal cofactor (as in Fig. 1d). Scale bar, 75 µm. Raw fluorescence intensity is shown on the top, and FRET ratio is shown on the bottom. Representative movies for cells stimulated without the retinal cofactor, unstimulated cells, and for stimulation of cells with the full optogenetic system are shown side-by-side.

File name: Supplementary Movie 2

Description: Localized optogenetic stimulation can drive a chemotaxis-like response (as in Fig. 1e). A representative cell stimulated with localized UV light pulses at the right edge of the cell is shown. Raw fluorescence intensity is shown on the left, and FRET ratio is shown on the right. The purple circle indicates the location of laser light stimulation. Scale bar, 25 µm. Color bar represents Cdc42 activity.

File name: Supplementary Movie 3

Description: Cdc42 activity is dose-dependent on receptor input strength (as in Fig. 2b). Scale bar, 75 µm. Representative movies for populations of cells stimulated with different light intensities are shown side-by-side. Raw fluorescence intensity is shown on the top, and FRET ratio is shown on the bottom for each condition.

File name: Supplementary Movie 4

Description: Cdc42-KO cells engage in many repolarization events, including building large, unstable lamellipodia. Scale bar, 15 µm. Movies of a representative control cell and Cdc42-KO cells were appended.

File name: Supplementary Movie 5

Description: Cdc42-KO cells demonstrating the cytoplasmic tether phenotype; the cell front pulls away from the cell body, but remains attached via a thin cytoplasmic tether. Scale bar, 15 µm.

File name: Supplementary Movie 6

Description: Cdc42 spatial response to local, stimulation in the cell center (as in Fig. 7a). A single, 4.3 µW, 10 ms laser pulse was applied at t = 0 s. Purple circle indicates the stimulation site. Color bar represents Cdc42 activity. Scale bar, 25 µm.
