# Supplementary material for: Optogenetic control of receptors reveals distinct roles for actin- and Cdc42-dependent negative signals in chemotactic signal processing
Source: Nat Commun. 2021 Nov 16;12:6148. doi: 10.1038/s41467-021-26371-z (PMC8595684; doi:10.1038/s41467-021-26371-z)
Supplement: Supplementary file 9 — Reporting Summary [file 41467_2021_26371_MOESM9_ESM.pdf]

nature research

Corresponding author(s): Sean R. Collins

Last updated by author(s): Sep 15, 2021

Reporting Summary

Nature Research wishes to improve the reproducibility of the work that we publish. This form provides structure for consistency and transparency in reporting. For further information on Nature Research policies, see our Editorial Policies and the Editorial Policy Checklist.

Statistics

For all statistical analyses, confirm that the following items are present in the figure legend, table legend, main text, or Methods section.

n/a	Confirmed	
☐	☒	The exact sample size (*n*) for each experimental group/condition, given as a discrete number and unit of measurement
☐	☒	A statement on whether measurements were taken from distinct samples or whether the same sample was measured repeatedly
☐	☒	The statistical test(s) used AND whether they are one- or two-sided<br>*Only common tests should be described solely by name; describe more complex techniques in the Methods section.*
☒	☐	A description of all covariates tested
☐	☒	A description of any assumptions or corrections, such as tests of normality and adjustment for multiple comparisons
☐	☒	A full description of the statistical parameters including central tendency (e.g. means) or other basic estimates (e.g. regression coefficient) AND variation (e.g. standard deviation) or associated estimates of uncertainty (e.g. confidence intervals)
☐	☒	For null hypothesis testing, the test statistic (e.g. F, t, r) with confidence intervals, effect sizes, degrees of freedom and P value noted<br>*Give P values as exact values whenever suitable.*
☒	☐	For Bayesian analysis, information on the choice of priors and Markov chain Monte Carlo settings
☒	☐	For hierarchical and complex designs, identification of the appropriate level for tests and full reporting of outcomes
☒	☐	Estimates of effect sizes (e.g. Cohen's *d*, Pearson's *r*), indicating how they were calculated

Our web collection on statistics for biologists contains articles on many of the points above.

Software and code

Policy information about availability of computer code

Data collection	1. Fluorescence microscopy images were acquired using Micro-manager version 1.4.23<br>2. Microscopy experiments were automated using custom code written in Matlab versions 2015a (Mathworks). The procedures executed by the code is described in the Methods, and code will be available upon request to the corresponding author.<br>3. Western blot images were collected using a Li-Cor Odyssey Imager (model 9120), and were contrast adjusted and cropped using FIJI (ImageJ) version 2.0.0-rc-43/1.53g.
Data analysis	Data was analyzed using custom code written in Matlab version 2020a (Mathworks). The procedures executed by the code is described in the Methods, and the code has been deposited at https://github.com/srcollins/Code-from-Bell-et-al-2021.

For manuscripts utilizing custom algorithms or software that are central to the research but not yet described in published literature, software must be made available to editors and reviewers. We strongly encourage code deposition in a community repository (e.g. GitHub). See the Nature Research guidelines for submitting code & software for further information.

Data

Policy information about availability of data

All manuscripts must include a data availability statement. This statement should provide the following information, where applicable:

- Accession codes, unique identifiers, or web links for publicly available datasets
- A list of figures that have associated raw data
- A description of any restrictions on data availability

The amplicon sequencing data generated in this study have been deposited in the NCBI-Trace sequence read archive under the BioProject accession code PRJNA720484PRJNA720484 [https://www.ncbi.nlm.nih.gov/bioproject/PRJNA720484/]. Control sequences (BioSample accession code

SAMN18651945SAMN18651945 [https://www.ncbi.nlm.nih.gov/biosample/18651945]) and Cdc42-KO sequences (BioSample accession code SAMN18651944SAMN18651944 [https://www.ncbi.nlm.nih.gov/biosample/18651944]) are stored in separate files. All other processed data generated in this study are provided in the Supplementary Information and Source Data file. Due to the large size of the full data set, raw images are not included but are available upon reasonable request. Source data are provided with this paper.

Field-specific reporting

Please select the one below that is the best fit for your research. If you are not sure, read the appropriate sections before making your selection.

☒ Life sciences ☐ Behavioural & social sciences ☐ Ecological, evolutionary & environmental sciences

For a reference copy of the document with all sections, see nature.com/documents/nr-reporting-summary-flat.pdf

Life sciences study design

All studies must disclose on these points even when the disclosure is negative.

Sample size	No sample size calculations were performed. Sample sizes were chosen based on practical limitations, but were chosen to exceed those that were sufficient to resolve effects in similar experiments from published studies (Yang et al, Nat Cell Biol, 2015. PMID:26689677; O'Neill et al, Dev Cell, 2018. PMID: 29937389).
Data exclusions	Dead cells were removed from analysis based on observable characteristics including autofluorescence. For the analysis in Figure 5, fast moving cells were removed to allow unambiguous comparison of before and after images. The exclusion criteria were based on analysis of a subset of control cells and were established before analysis of any perturbations. All exclusions applied are described in the methods section.
Replication	All results were replicated in at least three independent experiments. All findings reported were replicated on all attempts.
Randomization	Samples were allocated into experimental groups randomly, based on alternating execution of conditions to be compared in order designed to minimize temporal differences in execution of experiments for different conditions.
Blinding	All imaging protocols were automated such that experimenters were blind to the samples during experiments. Analyses were automated such that experimenters were blind to the sample groups for all experiments except for those shown in Figure 4d-f. For those experiments, the tether phenotype was scored manually, but this phenotype is extremely distinctive, and the analysis only involved counting numbers of cell exhibiting or not exhibiting the phenotype.

Reporting for specific materials, systems and methods

We require information from authors about some types of materials, experimental systems and methods used in many studies. Here, indicate whether each material, system or method listed is relevant to your study. If you are not sure if a list item applies to your research, read the appropriate section before selecting a response.

Materials & experimental systems

n/a	Involved in the study	
☐	☒	Antibodies
☐	☒	Eukaryotic cell lines
☒	☐	Palaeontology and archaeology
☒	☐	Animals and other organisms
☒	☐	Human research participants
☒	☐	Clinical data
☒	☐	Dual use research of concern

Methods

n/a	Involved in the study	
☒	☐	ChIP-seq
☒	☐	Flow cytometry
☒	☐	MRI-based neuroimaging

Antibodies

Antibodies used	Recombinant Rabbit monoclonal [EPR15260] to Cdc42. Abcam, Cat# ab187643. Lot # GR3207205-1 (1.307 mg/ml) YP010508PS; Rabbit anti-β-actin polyclonal antibody. Cell Signaling Technologies, Cat# 4967s, Lot # 09; IRDye 800CW Donkey anti-Rabbit IgG secondary antibody. Li-Cor, Cat# 925-32213, Lot# C91112-07.
Validation	Cdc42 antibody was knock-out validated for western blots by the manufacturer. The antibody detects a Cdc42 specific band that is ~21 kDa. Beta Actin antibody was validated for western blots by the manufacturer using the following approaches: 1) checking specificity/cross reactivity by testing several cell types. 2) Using Knock-out lines to verify specificity. The antibody recognizes a β-actin specific band that is ~45kDa. The anti-Rabbit antibody was validated for specificity to rabbit IgG heavy and light chains using ELISA and solid-phase adsorption assays and is recommended for western blots by the manufacturer.

Eukaryotic cell lines

Policy information about cell lines

Cell line source(s)	PLB-985 cells were originally obtained from the lab of Orion Weiner. HEK-293T cells were obtained from the ATCC.
Authentication	PLB-985 cells have highly characteristic behaviors, including rapid migration, elongated morphology, and responsiveness to formyl peptides. Cell lines were confirmed based on behavior, and analysis of RNA-seq data. PLB-985 and HEK-293T cells were also authenticated by STR profiling.
Mycoplasma contamination	Cell lines were tested prior to disposal for mycoplasma to confirm the lack of contamination. All cell lines used in the study tested negative.
Commonly misidentified lines (See ICLAC register)	PLB-985 is known to be a misidentified cell line that is actually a sub-line of HL-60 cells. We confirmed this by analysis of SNPs. We chose to use this sub-line because we observed better migration in under agarose conditions. We have noted in the Methods section that PLB-985 is actually a sub-line of HL-60, and that we have confirmed this directly.
